# Mandatory environmental and human rights due diligence

**DOI:** 10.18332/tid/133750

**Published:** 2021-03-22

**Authors:** Danielle van Kalmthout, Kelsey Romeo-Stuppy, Laurent Huber, Sonja von Eichborn, Claire Clément

**Affiliations:** 1 Belgian Alliance for a Smoke-Free Society, Brussels, Belgium; 2 The Human Rights Centre, Ghent University, Ghent, Belgium; 3 Action on Smoking and Health, Washington, United States; 4 BLUE 21, Berlin, Germany; 5 Unfairtobacco, Berlin, Germany; 6 Smoke Free Partnership, Brussels, Belgium

**Keywords:** tobacco control, human rights, environment, due diligence

The European Green Deal aims at getting Europe on track to achieve the Sustainable Development Goals (SDGs) that are based on human rights and the planetary boundaries. Tobacco is thwarting sustainability in all stages of the production and consumption chain. Tobacco use causes 8 million deaths per year and is the single most preventable risk factor for all non-communicable diseases (NCDs). Therefore, the WHO Framework Convention on Tobacco Control (WHO FCTC) is specifically mentioned as the prime instrument to achieve SDG 3, namely as Target 3.A. At the same time, human rights violations and environmental destruction are rampant in the supply chain of tobacco while tobacco corporations ignore their responsibility to act upon these in a significant way.

In 2021, the European Commission will introduce a proposal for legislation on mandatory human rights and environmental due diligence for companies as part of the Commission’s 2021 work plan and the European Green Deal^1^ which highlighted that sustainability should be ‘further embedded into the corporate governance framework, as many companies still focus too much on short-term financial performance compared to their long-term development and sustainability aspects’. The EU plays a leading role in the world when it comes to environmental protection and human rights. By adopting an ambitious mandatory due diligence legislation, the EU could confirm its leadership.

## What impact have tobacco products on the environment and human rights?

### Environment

The environmental impact of the tobacco industry has been described in detail in a study of the WHO/FCTC ‘Cigarette smoking. An assessment of tobacco's global environmental footprint across its entire supply chain, and policy strategies to reduce it’^2^:

‘Cigarette production and consumption have seen dramatic growth in recent decades and although the health effects of smoking are widely recognized, its impacts on the environment are largely overlooked. From tobacco cultivation^3,4^ and curing, to cigarette manufacturing, distribution, consumption and discarding, every stage in the global tobacco supply chain involves considerable resource inputs, and results in the production of wastes and emissions. Consequently, tobacco puts pressure on the planet's already stressed natural resources and its fragile ecosystems, threatening the livelihoods and future development of communities around the world^2^. The environmental damage that tobacco causes, on top of its negative health, social and economic impacts, makes it incompatible with the global development agenda. Reducing and ultimately eliminating cigarette production and consumption should be an integral part of strategies to achieve the Sustainable Development Goals (SDGs) (including goals 12, 13, 14, and 15).’

Tobacco butt pollution is also an essential issue to consider. About 4.5 trillion cigarettes^5^ are discarded each year worldwide, amounting to 80 million kg of tobacco waste and making cigarette butts the most littered item on Earth^6^. Cigarette butts are toxic to animals and children that may swallow them, they pollute groundwater, and they leach chemicals into the soil. Compounding this problem is the waste from other items related to smoking such as cigarette packages and lighters or matches. Cigarette butts and other tobacco-related wastes are a massive environmental problem.

The ecological footprint of tobacco is comparable to that of entire countries. Globally, the tobacco supply chain contributes about 84 Mt CO2 equivalent in emissions, e.g. equaling the combined footprint of Denmark, Luxembourg, Latvia and Lithuania^7^.

Next to traditional cigarettes, e-cigarettes cause environmental damage. From mining to manufacturing, using, and disposing, each stage of the e-cigarette product lifecycle presents novel environmental harms comparable to traditional cigarettes^8^. Tobacco companies already recognize that e-cigarettes pose new environmental burdens, necessitating them to ‘manage new areas of impact due to the increasing use of electronics and batteries in [their] products’^8^.

‘Improper disposal of e-cigarettes and e-liquid products can hurt the environment. If thrown in the trash or flushed into the sewer system, the nicotine solution in an e-liquid product can seep into the ground or water and become a danger for wildlife and humans. As e-cigarette batteries degrade, the compounds in them can also seep into nearby water. Additionally, lithium-ion batteries have been linked to explosions in recycling trucks when batteries are not properly disposed of.’^9^

The tobacco industry ignores its responsibility when it comes to the environmental costs on ecosystems, humans, flora and fauna of their business. These companies have promoted policies that avoid all environmental responsibility of the producer, and they attempt to divert public attention away from their environmental responsibilities through corporate social responsibility programmes^10^. The external environmental costs are borne by society and low-and middle-income countries in particular^11^.

### Human rights

Tobacco smoke and exposure to secondhand smoke together kill more than 8 million people each year^12^. To address this global epidemic, the Framework Convention on Tobacco Control (FCTC, joined by 182 Parties) was developed ‘to protect present and future generations from the devastating health, social, environmental and economic consequences of tobacco consumption and exposure to tobacco smoke’^13^.

Besides the general right to life and the right to health, tobacco products:

- Violate children’s rights^14^, including their protection from child labor in tobacco production^15,16^ (child labor in tobacco growing has been defined as one of the worst forms of child labor due to its detrimental effect on the physical and mental health of the children, especially from nicotine poisoning) and from misleading information (e.g. advertising);
- Violate women’s rights^17^, including protection from the impact of (passive) smoking on pregnancy, and adversely impact the rights of other vulnerable populations, such as the LGBT community, racial minorities, and indigenous populations, largely through targeted advertising^18^.

The impact of tobacco products on human rights has been noted in a number of human rights fora, directly and implicitly. The Committee on Economic, Social and Cultural Rights, in its General Comment No. 14, stated that the ‘failure to discourage production, marketing and consumption of tobacco’ constitutes a violation of the obligation to protect under Article 12 of the International Covenant on Economic, Social and Cultural Rights, mirroring language in the FCTC Chapeau. Likewise, General Comment 15 of the Committee of the Rights of the Child noted that governments must implement and enforce the FCTC as part of their obligations under the Convention on the Rights of the Child^19^.

There are also examples of human rights treaty bodies replying directly to countries about the impacts of tobacco products. In 2020 for example, in its concluding observations, the Committee on the Elimination of all forms of Discrimination Against Women (CEDAW) expressed concern about the negative impacts of tobacco on the women of Argentina, particularly about tobacco advertising directed at women. The Committee went on to urge Argentina to ratify and implement the FCTC.

After conducting a human rights assessment for a multinational tobacco company, the Danish Institute for Human Rights concluded:

‘According to the UNGPs companies should avoid causing or contributing to adverse impacts on human rights. Where such impacts occur, companies should immediately cease the actions that cause or contribute to the impacts. Tobacco is deeply harmful to human health, and there can be no doubt that the production and marketing of tobacco is irreconcilable with the human right to health. For the tobacco industry, the UNGPs therefore require the cessation of the production and marketing of tobacco.’^20^

The link between environment and human rights in tobacco cultivation is rightfully included in the FCTC’s Article 18 ‘Protection of the environment and the health of persons’ which stipulates that ‘In carrying out their obligations under this Convention, the Parties agree to have due regard to the protection of the environment and the health of persons in relation to the environment in respect of tobacco cultivation and manufacture within their respective territories’.

The link has also been recognized by countries that include the environment in their national action plans (NAPs) on human rights, for example, Belgium, Denmark, France, Germany, Poland, and many others^21^.

## Why do we need a mandatory environmental and human rights due diligence initiative?

Non-binding environmental and human rights due diligence exists at international level. In 1976, the non-binding OECD Guidelines for Multinational Enterprises were adopted and were revised for the last time in 2011^22^. The OECD also developed sector-specific due diligence schemes, such as for garment and footwear supply chains. The UN Guiding Principles on Business and Human Rights (UNGP)^23^ were endorsed by the Human Rights Council in its resolution 17/4 of 16 June 2011. UNICEF launched the Children’s Rights and Business Principles (CRBPs) together with the UN global Compact^24^ and Save the Children in 2012. The international guidelines are addressed to State Parties and not directly to companies.

Corporate Social Responsibility (CSR) is a crucial part of due diligence, as companies need to ensure that their suppliers are also meeting all the CSR commitments. In a globalizing world, tobacco corporations, just like many other multinational companies, have adapted to public expectations deriving from these initiatives and developed CSR schemes on a voluntary basis^25^. In tobacco growing countries such as Brazil, India, Indonesia or Malawi, tobacco companies fund programs that aim to mitigate the environmental losses due to tobacco^26^. Furthermore, all major multinational tobacco corporations fund the Eliminating Child Labour in Tobacco Growing Foundation^27^ which aims at alleviating poverty, freeing children from child labor and supporting their education. As well, tobacco corporations included the narrative of sustainable development into their corporate strategies^28^.

Although these voluntary CSR schemes exist, it is clear that their scope is completely insufficient to reverse the dramatic environmental impact of tobacco production and consumption or to stop the continuing violation of human rights^29^, as described above^26^. These voluntary schemes are also used as part of communication strategies to portray the companies’ practices under the best light^30^. Voluntary disclosure results in environmental impact data that is vague, unclear and inconsistent in its coverage and methodologies^26^. This creates several problems. There is no industry-wide standardized format, which makes it difficult to track progress or make comparisons between companies. New units of measurements were created able to obscure the true scale of the environmental impact. Companies are free to set their own goals and choose to disclose on topics that portray their practices in the best light.

Tobacco companies not only use voluntary CSR schemes to improve their public image but also to weaken and undermine tobacco control policies by preventing the introduction of legally enforceable tobacco control measures which have a proven record of effectiveness in reducing tobacco consumption^10^. By taking broad-based but effective action against the environmental hazards created by the tobacco industry, the demand for tobacco products will be further reduced. With strengthened environmental policies, there will be increased costs for tobacco products, decreased social acceptance of tobacco use and changes in the most commonly used tobacco products^31^. According to WHO, tobacco companies use CSR to compromise or to propose voluntary agreements that would obviate the need for legislation or regulation^25^.

‘Research and experience have shown, however, that voluntary agreements and compromises with the industry do not translate into public health gains. Therefore, the tobacco industry's proposal to substitute self-regulation for government regulation is essentially ineffective; governments are more effective in tobacco control when they do not endorse voluntary codes of conduct or self-monitoring by the tobacco industry and do not accept assistance from or direct consultation with the tobacco industry on appropriate language for tobacco control legislation or other legal instruments (apart from legitimate forums, such as public hearings and written submissions).’^25^

The tobacco industry is therefore an excellent example to emphasize that voluntary CSR schemes do not advance human rights and environmental protection, but serve as a tool to continue making profits without conscience. Thus, a mandatory due diligence framework is needed to achieve the SDGs and to protect and fulfil human rights.

Different Member States within the European Union have taken legislative initiatives or are in the process of developing due diligence legislation. In not less than 11 EU Members States (and Norway, Switzerland) initiatives towards due diligence laws have been discussed or adopted^32^. Some examples are: the Netherlands adopted in 2019 the Dutch Child Labour Due Diligence Act to prevent child labor being used in goods and services brought to the Dutch market; the French Duty of Vigilance Law, adopted in 2017, wants to identify risks and prevent human rights and environmental impact, and requires companies to publish a ‘vigilance plan’; and Germany is in the process of drafting a law on human rights and environmental due diligence following strong pressure from civil society.

Just as voluntary CSR schemes fail to achieve their objectives, academic research has shown that voluntary corporate tools that implement due diligence have not been sufficiently effective at securing respect for rights^33^.

Taking this into account, we are of the opinion that a European mandatory due diligence on environment and human rights, which explicitly includes the tobacco industry, is a necessary step to achieve sustainable development as it would ensure:

- That one binding framework is implemented in a coordinated way over the whole of the EU, while leaving the implementation to the Member States;
- A level playing field for companies operating in the EU;
- Companies are addressed directly (not via State Parties) as well;
- Solutions to mitigate the environmental impact of tobacco products;
- Responsibility is placed on the tobacco companies as a polluter;
- A contribution from companies to the overall solution to achieve the Sustainable Development Goals.

When it comes to the tobacco industry, some challenges to make due diligence a success have to be taken into account:

- The lack of robust data on and awareness of tobacco’s true environmental cost among smokers, general public, and even policy-makers;
- The differences in national regulations being exploited by tobacco companies to avoid reporting or paying for the damage caused by their activities;
- Dependence on tobacco as a cash crop in a number of lower income countries;
- Strong tobacco lobby and the growing uptake of smoking, particularly in low- and middle-income countries^2^.

## What have tobacco control NGOs done towards due diligence up to now?

Due diligence has been at the forefront of the work of tobacco control advocates since the negotiations on the WHO Framework Convention on Tobacco Control in the early 2000s. The advocacy of civil society groups led to the Convention explicitly referring to the supply chain in Article 18: ‘In carrying out their obligations under this Convention, the Parties agree to have due regard to the protection of the environment and the health of persons in relation to the environment in respect of tobacco cultivation and manufacture within their respective territories’.

Tobacco control advocates have been working to highlight connections between tobacco control and human rights for years, and have made significant progress. Due to the efforts of civil society, tobacco control and the goals of the FCTC were included as a target in the Sustainable Development Goals, goals that are grounded in human rights^34^. In 2017, after discussions with tobacco control groups, the Danish Institute for Human Rights (DIHR) concluded that: ‘There can be no doubt that the production and marketing of tobacco is irreconcilable with the human right to health’^35^. In 2018, the World Conference on Tobacco or Health (WCTOH) adopted an exemplary Cape Town Declaration supported by 165 organizations and formally submitted to the Office of the High Commissioner for Human Rights^18^. Similar declarations were adopted by civil society at conferences in Madrid and Bucharest^36^.

Civil society has been working to include human rights and due diligence in domestic tobacco control efforts as well. In May 2020 for example, an alliance of 19 civil society organizations working on tobacco control, public health, sustainable development and children’s rights jointly submitted the alternative report ‘Children’s Rights and Tobacco Control in Germany’ to the UN Committee for the Rights of the Child during the 5th/6th reporting cycle for Germany^37^. Additionally, school children were supported to submit their own report ‘We want tobacco to stop being sold’ also referring to child labor in the tobacco supply chain^38^.

Early in 2020, an alliance of 10 civil society organizations submitted the report ‘Tobacco control in Germany: Failure to protect the right to health and women's rights in supply chains’ to the UN Committee on the Elimination of Discrimination Against Women. This report led to the CEDAW asking the German government about their legislation to hold corporations accountable for women’s rights violations in their operations abroad^39^.

## What are key issues for an EU mandatory environmental and human rights due diligence?

### General scope

The future EU law should include all global human rights treaties and cover all types of violations, including specific human rights conventions such as UN Convention on the Rights of the Child (CRC), the Convention on Elimination of All Forms of Discrimination Against Women (CEDAW), the Convention on the Right of Persons with Disabilities (CRPD), the International Convention on All Migrant Workers and Members of their Families (ICRMW), the Indigenous and Tribal People Convention (ILO 169), the UN Declaration on the Rights of Indigenous Peoples (UNDRIP), the International Covenant on Economic, Social, and Cultural Rights (ICESCR) and the International Convention on the Elimination of All Forms of Racial Discrimination (CERD).

All companies have the responsibility to protect the environment and respect human rights. The legislation should cover all companies – either domiciled in an EU Member State or placing products or providing services in the internal market – regardless of their size and take a non-sector specific approach^40^. The scope of obligations should be differentiated by criteria such as the companies’ risk profile and their annual turn-over.

### Specific rights example – the rights of the child

It is necessary that the mandatory due diligence legislation explicitly refers to additional measures for vulnerable groups, such as children. The UN CRC should be referred to as a minimum standard for businesses to respect, keeping in mind that the UN CRC has been ratified by almost all countries in the world.

Children and adolescents are particularly vulnerable to the effects of tobacco production and consumption. The widespread use of child labor in connection with the living and working conditions in tobacco cultivation specifically violates children’s rights to: health (UN CRC Art. 24); adequate standard of living (UN CRC Art. 27); education (UN CRC Art. 28); leisure (UN CRC Art. 31); and protection from economic exploitation (UN CRC Art. 32). Both the marketing of addictive and harmful tobacco products, which is specifically targeted at children and adolescents, and the lack of protection from secondhand smoke, violate children’s rights to: life (UN CRC Art. 6), information (UN CRC Art. 17), health (UN CRC Art. 24) and protection from narcotic drugs (UN CRC Art. 33). In 2013, the UN Committee on the Rights of the Child published its General Comment on the Right to Health and explicitly referred to the need to transpose the WHO Framework Convention on Tobacco Control into domestic law^14^.

The UN Guiding Principles on Business and Human Rights (UNGP) have very little reference to children. The commentary of Principle 12 states that ‘depending on circumstances, business enterprises may need to consider additional standards’ and implicitly refers to the UN CRC by mentioning the United Nations instruments on the rights of children. However, the UNGP does not explicitly refer to the CRC as a minimum standard for businesses to respect.

Likewise, the OEDC guidelines use in chapter IV on Human Rights the same conditional wording^22^ as the UNGP. Besides the general guidelines, sector specific due diligence guidance exists for different business sectors. Concerning the worst forms of child labor, the OECD has developed a guidance only for companies with a mineral supply chain^41^.

### Implementation

It should be highlighted that the tobacco industry conducts activities described as socially responsible to distance its image from the lethal nature of the product that it produces and sells or to interfere with the setting and implementation of public health policies. Activities that are described as ‘socially responsible’ by the tobacco industry, aiming at the promotion of tobacco consumption, is a marketing as well as a public relations strategy that falls into the definition of advertising, promotion and sponsorship of the FCTC. According to WHO, corporate social responsibility of the tobacco industry is an inherent contradiction, as the industry’s core functions are in conflict with the goals of public health policies with respect to tobacco control. It should be avoided at all times and it is expected that this EU initiative on due diligence will be used by tobacco corporations to polish their image. In that respect, the strict application of Art. 5.3 FCTC is decisive. This article states that Parties to the Convention should protect their public health policy against the commercial interests of the tobacco corporations.

### Identify and assess companies’ risks

Due diligence should require from the company and the partners within the supply chain, services and business partners, to identify the risks to human rights and prevent the environmental impact of the whole life cycle of their product. For the tobacco industry, the life cycle impacts of tobacco can be roughly divided into five key stages: 1) growing and curing; 2) product manufacture; 3) distribution and transportation; 4) product consumption, including secondhand and thirdhand smoke exposure; and 5) post-consumption tobacco product waste disposal^2^. Due diligence concerns stages 1 to 3. For each of these three stages a list of possible risks should be established in cooperation with stakeholders leading to a clear understanding of the environmental harm and human rights violations caused by tobacco production and consumption. Clear guidance and criteria are needed.

Once the risks are defined and assessed, the company should reverse the impact of these risks by ceasing, preventing or mitigating them within their company but also throughout the whole supply chain, services and business partners. This should be an ongoing process and clearly reported and communicated in all transparency.

### Monitoring

The EU mandatory ENV and HR DD should go beyond the simple obligation for companies to report on the steps that a company has taken (or not). It is highly recommended that the report should include a set of EU harmonized criteria to make sure that a minimum level of accurate information is published, in all transparency, and that this accurate information is comparable between companies and sectors. As well, the report should include a plan of action on how to reverse the impact of the risks.

An example is the ‘vigilance plan’ as integrated in the French Due Diligence Law^42^:

1. Une cartographie des risques destinée à leur identification, leur analyse et leur hiérarchisation;
2. Des procédures d’évaluation régulière de la situation des filiales, des soustraitants ou fournisseurs avec lesquels est entretenue une relation commerciale établie, au regard de la cartographie des risques;
3. Des actions adaptées d’atténuation des risques ou de prévention des atteintes graves;
4. Un mécanisme d’alerte et de recueil des signalements relatifs à l’existence ou à la réalisation des risques, établi en concertation avec les organisations syndicales représentatives dans ladite société;
5. Un dispositif de suivi des mesures mises en œuvre et d’évaluation de leur efficacité.

The Dutch Child Labour Due Diligence Act also goes beyond simple reporting and states in Art. 5.1 that:

‘If the subject company has a reasonable suspicion of child labor in the production of the goods or services, it must adopt and implement a plan of action. A joint action plan aimed at ensuring that affiliated companies exercise due diligence that is developed by or among one or more social organizations, employees' organizations or employers' organizations and approved by the Minister for Foreign Trade and Development Cooperation will satisfy this requirement.’^43^

### Enforcement and sanctions

An absolute necessary addition to companies identifying, assessing, preventing, mitigating, monitoring and reporting in transparency on potential and actual environmental impact and human rights violations are enforcement and sanctions where victims receive remedy. Sanctions may include administrative fines (in proportion to the company’s annual turn-over), civil liability and in the case of severe violations even criminal liability, as stated in the FCTC Article 19. The burden of proof and other possible barriers on access to justice should be minimized. Thus a set of different implementation mechanisms, including administrative, civil and possibly even criminal law instruments together with sanctions in requiring states to adopt approaches need to be included that will result in penalties sufficient to have a deterring effect. In any event, Member States would be required to implement the HRDD legislation effectively in accordance with generally accepted principles of EU law.

Local civil society can play a key role in the implementation and monitoring of such legislation, and there must be channels for the civil societies to interact with the authorities and provide feedback.

## Key messages

1. Mandatory human rights and environmental due diligence for companies is absolutely necessary to achieve the Sustainable Development Goals, to protect and fulfil human rights obligations and avoid environmental destruction.
2. The tobacco industry has an ecological footprint which is comparable to that of entire countries. The human rights violations include the worst form of child labor in tobacco growing, according to ILO.
3. Mandatory human rights and environmental due diligence should include transparent reports on standardized criteria, the obligation to develop and implement an ambitious plan of action, permanent monitoring structures, enforcement and sanctions.

## LIST OF SUPPORTING ORGANISATIONS

Advocacy Center for Life

Airspace Action on Smoking and Health

Alliance contre le tabac

Action on Smoking and Health- Washington, D.C.

ASH Finland

ASH Ireland, Council of the Irish Heart Foundation

ASH Scotland

Asian Consultancy on Tobacco Control

Austrian Council on Smoking & Health

Austrian medical chamber

Belgian Alliance for a Smoke Free Society

BLUE 21/Unfairtobacco

BlueLink Foundation

Cancer Research UK

Cancer Society of Finland

Catalan Institute of Oncology

CATR, Portugal

Center for public health support (Kazakstan)

Cigarette Butt Pollution Project

Comite National Contre le Tabagisme (CNCT)

Corporate Accountability

Corporate Accountability Lab

Cyprus National Addictions Authority (NAAC)

Danish Cancer Society

Department of Public Health and Social Medicine,

Medical University of Gdansk, Poland

Development and Salutat Astana (Kazakstan)

DNF - Pour un Monde Sans Tabac

European Medical Student Association (EMSA)

Εuropean Network for Smoking and Tobacco Prevention (ENSP)

EuroHealthNet

European Cancer Patient Coalition

European Chronic Disease Alliance

European Respiratory Society (ERS)

FCTC Implementation and Monitoring Center in Georgia

Fondation contre le cancer, Belgique

Healis Sekhsaria Institute for Public Health

Health Care Plus UG

Health Funds for a Smokefree Netherlands

Health Promotion Foundation (Poland)

Heart Foundation of Jamaica/Jamaica Coalition for

Tobacco Control

Heartland Initiative

Indraprastha Public Affairs Centre

Japan Society for Tobacco Control

Kenya Tobacco Control Alliance

Kosovo Advocacy & Development Centre (KADC)

Lithuanian Tobacco and Alcohol Control Coalition

Mexico Salud-Hable Coalition

Macedonian Respiratory Society

Nofumadores.org (Spain)

Norwegian Cancer Society

Progressive Reinforcement of Organizations and

Individuals (PROI)

Resource Centre For Primary Health Care

Romanian Society of Pneumology

SEATCA

Slovenian Coalition for Public Health, Environment and Tobacco Control

Smoke Free Israel

Smoke Free Life Coalition

Smoke Free Partnership (SFP)

Smoke-Free Bulgaria

Smokefree Kazakstan Coalition

Sociedad Uruguaya de Tabacologia

Stichting Rookpreventie Jeugd (Netherlands)

Suomen ASH/ASH Finland

TerraProject

The International Union Against Tuberculosis and Lung Disease

The Public Health Law Center

TobaccoFree Research Institute Ireland

University of Beira Interior, Faculty of Health Sciences (Portugal)

XQNS, Spain

Youth Network No Excuse Slovenia
